# Effects of Different Additives and Ratios on Broom Sorghum Straw Silage Characteristics and Bacterial Communities

**DOI:** 10.3390/microorganisms12102062

**Published:** 2024-10-15

**Authors:** Panjie Sheng, Baochao Bai, Mingjian Liu, Weiqin Ma, Jianliang Liu, Chaoran Song, Shuai Du, Gentu Ge, Yushan Jia, Zhijun Wang

**Affiliations:** 1 College of Grassland Science, Inner Mongolia Agricultural University, Hohhot 010019, China; 2 Key Laboratory of Forage Cultivation, Processing and High Efficient Utilization, Ministry of Agriculture, Hohhot 010019, China; shengpanjie@163.com (P.S.); bbc66688@163.com (B.B.); liumj_nm@163.com (M.L.); maweiqin0108@126.com (W.M.); liujianliang0202@163.com (J.L.); a1198494634@163.com (C.S.); dushuai_nm@sina.com (S.D.); gegentu@163.com (G.G.); 3 Key Laboratory of Grassland Resources, Ministry of Education, Hohhot 010019, China; 4 National Center of Pratacultural Technology Innovation (under Preparation), Hohhot 010010, China

**Keywords:** broom sorghum straw, cellulase, xylanase, silage performance, straw feedstuff

## Abstract

As a large agricultural country, China produces a large number of agricultural and sideline products while harvesting agricultural products every year. Crop straw is one of them. Broom sorghum is a traditional crop in China, which produces a large amount of straw resources every year. These straw resources are placed in the field and cannot be used efficiently. The purpose of this study was to solve the problem of straw utilization of Broom sorghum, one of the main food crops in arid and semi-arid areas of northern China. Broom sorghum is not only a nutritious food crop, its straw is also rich in crude fiber and mineral elements, which has high utilization value. However, due to the high content of lignocellulose in straw, the texture is hard, which limits its digestion and utilization efficiency as feed. In this study, the broom sorghum straw was used as the research object, and the straw raw materials were treated with *Lactobacillus plantarum*, cellulase and xylanase, respectively. After silage fermentation for 30 d and 60 d, the bags were opened to determine the nutritional quality, fermentation quality, microbial community structure and other indicators. The best fermentation time and additives for broom sorghum straw silage were comprehensively screened to improve the nutritional value of straw and animal production performance. The results showed that the nutritional quality of silage straw increased with the extension of fermentation time. Compared with silage straw after 30 days of fermentation, the nutritional quality and fermentation quality of straw were significantly improved after 60 days of fermentation. *Lactobacillus plantarum*, cellulase and xylanase could improve the silage performance of broom sorghum straw by improving the microbial community structure in straw, and the effect of cellulase was the best. When cellulase was used in straw at the standard of 20 U/g FM, the content of water-soluble carbohydrates could be significantly increased to 31.35 g/kg FM, and the concentration of lactic acid was also significantly increased to 23.79 g/kg FM. Therefore, in actual production, it is recommended to use cellulase at a dose of 20 U/g FM in broom sorghum silage and open the bag after 60 days of silage fermentation. The results of this study provided a scientific basis for the efficient utilization of broom sorghum straw as feed.

## 1. Background

Broom sorghum (*Sorghum bicolor var. technicum*) belongs to the genus Panicum of Gramineae, a one-year-old herbaceous plant. It is one of the main grain crops in arid and semi-arid areas of northern China and is also a typical grain and forage crop [1]. Broom sorghum has a high utilization rate of light, heat, water and soil resources, and has the characteristics of drought resistance, barren resistance, saline-alkali resistance and a short growth period. It is widely planted in China [2]. The broom sorghum grain is rich in starch, protein, fat, trace elements, minerals and polyphenols. It is an excellent food crop [3,4]. With the expansion of its planting area, the yield of straw has also increased. A large amount of straw is still in a simple and inefficient use state, such as forage grass reserves and firewood burning, and is even abandoned in the field [5]. The content of crude fiber in straw is high, and it is rich in mineral elements such as nitrogen, potassium, calcium and phosphorus. It has great utilization value and can be used as forage for herbivorous livestock [6].

Silage is an effective measure for the utilization of straw feed [7]. Studies have shown that adding microorganisms to crop straw can be prepared into a straw feed with good palatability through anaerobic fermentation [8]. After straw silage fermentation, its texture became soft, bright and juicy in color, accompanied by an acidic aroma, which could promote animal appetite and increase animal feed intake, and the fermented feed was sealed for a long time [9,10]. Relevant reports have pointed out that various fungi and enzymes added in forage fermentation are conducive to the degradation of cellulose, hemicellulose and other substances in straw into small molecular polysaccharides or glucose, which improve the nutritional value of the forage and improve the production performance of livestock [11,12]. In the experiment of feeding goats with fermented straw, the results showed that microbial fermented straw could improve the production performance and meat quality of goats [13].

There is no doubt that straw has great potential for feed utilization. At present, most scholars pay more attention to the research on the feed utilization of corn straw but ignore the utilization of unconventional crop straw [14,15]. These straw resources contain high lignocellulose or special volatile substances, and livestock do not like to eat or even do not eat, which seriously restricts the digestion and utilization efficiency of sorghum straw by livestock [16]. 

In this study, *Lactobacillus plantarum*, cellulase and xylanase were used to treat sorghum straw for silage, and the effects of different microbial enzyme preparations on the silage quality and microbial community structure of sorghum straw after fermentation were investigated. The improvement effect of silage on the nutritional quality of sorghum straw was clarified, which provided a scientific theoretical basis for the feed utilization of sorghum straw, allowed for the development of high-quality forage modulation technology, and injected new vitality into the development of animal husbandry.

## 2. Materials and Methods

### 2.1. Silage Feed Preparation

The raw material of broom sorghum straw was taken from the straw after harvesting seeds in the autumn of 2022 in Lindong Town, Balin Left Banner, Chifeng City, Inner Mongolia Autonomous Region (43°57′ N, 119°21′ E). The local farmland was sown in April every year and harvested in mid-to-late September. During the period, the fertilizer was applied once at the seedling stage and irrigated once, and the fertilizer was not applied at any other time. The straw was crushed to 2–3 cm, the water content of the raw material was 15%, and the water was added. The water content of the raw material by controlled at about 60% by stirring evenly. The straw raw materials with uniform stirring were divided into six parts for the following treatment: (1) no additive control (CK); (2) adding commercial *Lactiplantibacillus plantarum* ((LP; Yidu Biological Technology Co., Ltd., Hohhot, Inner Mongolia, China; addition amount: 1 × 10^6^ cfu/g fresh material (FM)); (3) cellulase (CE; Macklin Biochemical Technology Co., Ltd. Shanghai, China; CE activity as 1 × 10^5^ U/g; addition amount: 1 × 10^5^ U/kg FM); (4) cellulase (CE activity as 1 × 10^5^ U/g; addition amount: 2 × 10^5^ U/kg FM); (5) xylanase (XE; Macklin Biochemical Technology Co., Ltd. Shanghai, China; XE activity as 1 × 10^5^ U/g; addition amount: 1 × 10^5^ U/kg FM); (6) xylanase (XE activity as 1 × 10^5^ U/g; addition amount: 2 × 10^5^ U/kg FM). In each treatment, the raw materials and additives were stirred evenly, and the polyethylene bags (35 cm × 40 cm) were filled with 500 g per bag and vacuum sealed. There were three replicates for each treatment, and the bags were opened at 30 d and 60 d, with a total of 36 bags (6 treatments × 3 replicates × 2 times). Silage fermentation was carried out at 20–30 °C, and the nutritional quality, fermentation characteristics and bacterial community were determined after opening the bag.

### 2.2. Microbial Counts, Nutritional Quality and Fermentation Parameters

Microbial counts were analyzed according to Bai et al. [17], and lactic acid bacteria were cultured and counted on an MRS medium. Aerobic bacteria were cultured and counted using a nutrient agar medium. Molds and yeasts were cultured and counted on potato dextrose agar medium. Coliform bacteria were cultured and counted on eosin methylene blue medium. The media used in the experiment were purchased from Guangdong Huankai Microbial Science Co., Ltd. (Guangzhou, Guangdong, China) and China Guangzhou Antai Technology Co., Ltd. (Guangzhou, Guangdong, China).

Fresh broom sorghum straw silage feed was taken, and 10 g of broom sorghum straw sample was mixed with 90 mL sterilized water, beaten and homogenized for 2 min to obtain organic acid liquid. The pH value was measured by an electrode pH meter (PHS-3C, INESA Scientific Instrument Co., Ltd., Shanghai, China). According to Cheng et al.’s method [18], the concentrations of organic acids, lactic acid (LA), acetic acid (AA), propionic acid (PA) and butyric acid (BA) were determined by the Agilent 1260 HPLC system (Agilent Technologies, Inc., Waldbronn, Germany) equipped with a refractive index detector (column: Carbomix^®^ H-NP5; Sepax Technologies, Inc., Newark, DE, USA; eluent: 2.5 mmol/L H_2_SO_4_, 0.5 mL/min; temperature: 55 °C). The ammonia nitrogen (NH_3_-N) content was determined by the phenol sodium hypochlorite colorimetric method [19].

Each group of samples was tested three times in parallel for chemical analysis. Fresh or silage materials were dried in a fan oven at 65 °C for more than 72 h to constant weight to analyze dry matter content [20]. The content of crude protein (CP) was determined by Dumas combustion nitrogen determination method [21]. Soluble carbohydrate content (WSC) was determined by anthrone colorimetry [22]. Acid detergent fiber (ADF) and neutral detergent fiber (NDF) were determined by an ANKOM A200i fiber analyzer (ANKOM Technology Corp., Fairport, NY, USA) according to the method of Van Soest [23].

### 2.3. DNA Extraction, PCR and Sequencing

DNA was extracted from fresh and silage samples of broom sorghum straw using the E.Z.N.A.R commercial sample DNA extraction kit (Omega Bio-tek, Norcross, GA, USA), according to the description of Huang [24]. Agarose gel electrophoresis (1%) and NanoDrop 2000 UV-visible spectrophotometer were used to measure the concentration and purity of extracted DNA, and (338F: 5′-ACTCCTACGGGAGGCAGCAG-3′; 806R: 5′-GGACTACHVGGGTWTCTAAT-3′) was used as a 16S rDNA primer to amplify the V3-V4 variable region by PCR. The library was built using the NEXTFLEX^®^ Rapid DNA-Seq Kit and sequenced using Illumina’s Miseq PE300 platform (Shanghai Meiji Biomedical Technology Co., Ltd., Shanghai, China).

### 2.4. Bacterial Community Sequencing Analysis

For the double-ended data obtained by sequencing, the sample data were split according to the barcode information, and the connector and barcode sequence were removed. FLASH (v1.2.8) was used to merge the paired sequences, and then, fqtrim (v0.94) was used to perform quality filtering on the original read data under specific filtering conditions to obtain a high-quality clean sequence. Next, we used Vsearch (v2.3.4) to filter chimeric sequences. QIIME2 (v2019.7) was used to import and process the sorted read data for bioinformatics analysis. Using the Divisive Amplicon Denoising Algorithm 2 (DADA2), we acquired the feature table and feature sequence after the dereplication. In order to calculate the Alpha diversity and β-diversity, identical sequences created at random were normalized. After that, on the basis of the SILVA (version 138) classifier, the relative abundance of each microorganism sample was used to normalize the feature abundance. FIn order was used to determine the species diversity of each sample under different treatments, and the q2-diversity plug-in of QIIME2 (v2019.7) was used for alpha and beta diversity analysis. Principal Coordinate Analysis (PCoA) using weighted UniFrac outputs was used to evaluate and visualize bacterial communities between individuals and groups. According to the method of Segata [25], LEfSe analysis (linear discriminant analysis effect size) (LDA > 3) was used to analyze the bacterial groups with significant differences in abundance from phylum to species level among different groups. The Mann–Whitney U test was used to identify differences in relative abundance among different populations. Random forest analysis was used to determine the key microorganisms in the process of broom sorghum straw silage. In order to clarify the interaction between microorganisms and fermentation quality, the mantel test correlation network heat map was used to visualize the relationship between key bacterial communities and fermentation quality. Phylogenetic Investigation of Communities by Reconstruction of Unobserved States 2 (PICRUSt2) was used to infer the functional pathways of broom sorghum straw silage microorganisms based on the information in the KEGG database [26]. A bar chart was drawn using the GraphPad Prism 9 software.

### 2.5. Data Analysis

Excel software. 2021 was used for data collation. Alpha diversity, chemical composition and fermentation characteristics were analyzed using SAS ver. 9.2 (SAS Institute, 2007 Cary, NC, USA). The statistical model of SAS is: Yij = μ + Ti + Dj + (T × D) ij + εij, where Yij = response variable, μ = overall mean, Ti = effect value of different treatments (CK, LP, CE10, CE20, XE10, XE20), Dj = effect value of silage time (30 d, 60 d), (T × D) ij = interaction effect value of different treatments and silage time, εij = random residual. The difference between the means was analyzed by analysis of variance (ANOVA) Tukey HSD post hoc test, and *p* < 0.05 was considered statistically significant.

## 3. Results and Analysis

### 3.1. Effects of Additives and Their Proportion on Nutritional Quality of Broom Sorghum Straw

#### Overview of Raw Materials of Broom Sorghum Straw

The nutrient composition of the straw raw material is shown in Table 1. From the composition point of view, sorghum straw contains higher ADF and NDF, while CP and WSC content is lower. The microbial community on the surface of sorghum straw was mainly composed of lactic acid bacteria, yeast, coliform bacteria, aerobic bacteria and mold. In terms of quantity, aerobic bacteria that were not conducive to silage fermentation occupied a dominant position, while lactic acid bacteria did not occupy a dominant position.

### 3.2. Nutritional Quality of Straw Silage

The dynamic changes in the nutritional quality of broom sorghum straw fermentation are shown in Table 2. Different treatments had significant effects on the nutritional indexes of sorghum straw, and silage time had significant effects on DM, CP, WSC and NDF (*p* < 0.05), and the nutritional quality of silage increased with the extension of silage time. After 60 days of ensiling, the nutritional components of sorghum straw with different additives were significantly different (*p* < 0.05). Compared with 30 d, the contents of DM, ADF and NDF in each treatment group showed a downward trend, and after 60 days of fermentation, the dry matter of CE20 and LP treatment, the ADF of CK, CE10 and CE20, and the NDF of CK, CE20 and XE20 changed most significantly. Among different treatments, the DM content of sorghum straw in LP, CE10 and CE20 treatment groups was significantly lower than that in CK treatment (*p* < 0.05), but the content of XE10 treatment was significantly higher than that of the CK treatment (*p* < 0.05). The CP content of broom sorghum straw showed an increasing trend with the extension of silage time, and XE10 and XE20 were significantly lower than that of other treatment groups (*p* < 0.05). From the time point of view, the extension of fermentation time is not conducive to the preservation of WSC content, and the WSC content in different treatments is higher than that of 60 d in 30 d. Among different treatments, the improvement effect of CK and LP on WSC content was significantly lower than that of the other four treatments (*p* < 0.05). This indicates that there is a significant interaction between silage time and additive treatment on CP and WSC content in straw.

### 3.3. Fermentation Characteristics of Straw Silage

The fermentation quality of sorghum straw treated for 30 days and 60 days is shown in Table 3. BA was not detected in all treatments. Different treatments had significant effects on the fermentation indexes of sorghum straw, and silage time had a significant effect on PA. The pH and NH_3_-N of different treatments almost did not change with the increase in silage days. Except for CK and CE10, the NH3-N of other treatments showed a stable state. The PA of CK, LP, CE20 and XE20 straw increased significantly with the increase in fermentation days (*p* < 0.05). The results of different treatments showed that the pH effect of xylanase treatment was better than that of other treatments. The contents of LA and AA in cellulase treatment were significantly higher than those in other treatments. The addition of enzyme preparation could significantly reduce the AA and PA of silage sorghum straw (*p* < 0.05). Compared with CK, the content of NH_3_-N in other treatments was significantly lower, indicating that the use of exogenous additives was beneficial to the retention of crude protein in straw silage. In addition, according to the results, the interaction between different treatments and silage time was the most significant in NH3-N and PA.

### 3.4. Bacterial Diversity and Community Composition of Broom Sorghum Straw Silage 

#### Microbial Community Structure of Broom Sorghum Straw Silage

**Figure 1:**
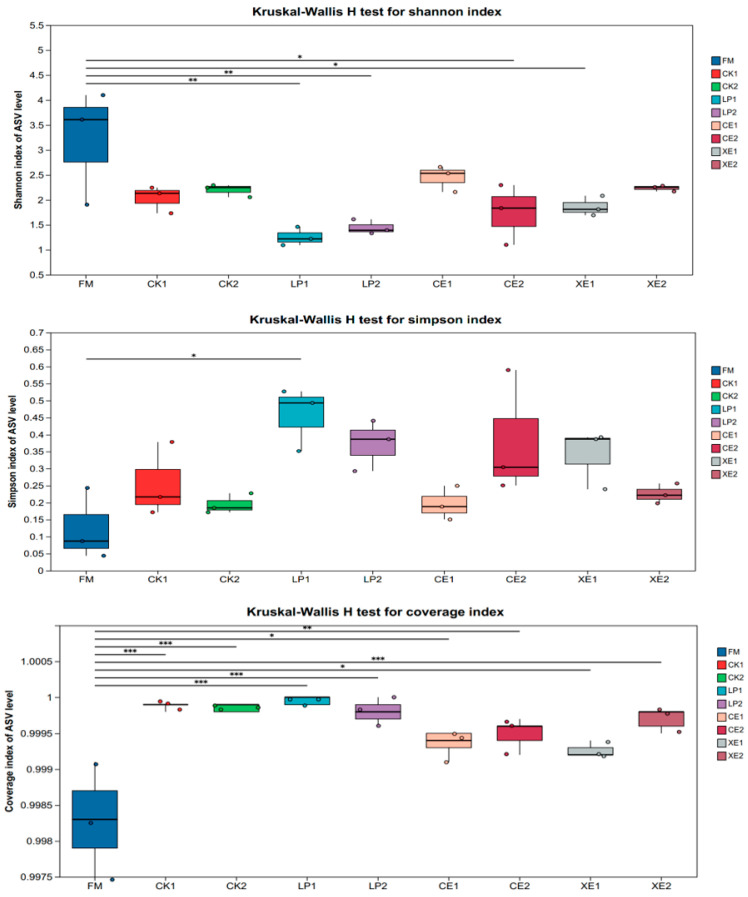
Effects of different additives and ensiling days on α diversity of broom sorghum straw silage. This figure shows the significant differences between the selected two groups of samples and marks the two groups with significant differences (0.01 < *p* ≤ 0.05 marked as *, 0.001 < *p* ≤ 0.01 marked as **, *p* ≤ 0.001 marked as ***). The abscissa is the grouping name, and the ordinate is the index size of each group. FM: fresh matter; CK1: CK fermentation for 30 days; CK2: CK fermentation 60 days; LP1: LP fermentation for 30 days; LP2: LP fermentation for 60 days; CE1: CE20 fermentation for 30 days; CE2: CE20 fermentation for 60 days; XE1: XE20 fermentation for 30 days; XE2: XE20 fermentation for 60 days. The same for both figures.

Combined with the analysis of the results of nutritional indicators and fermentation indicators of different treatments, this study did not conduct microbial research on CE10 and XE10. The microbial community alpha diversity of broom sorghum straw silage after 30 d and 60 d under different treatments is shown in Figure 1. The use of different additives significantly affected the microbial community diversity (Shannon) of sorghum straw silage, but the silage time had no significant effect on each index (*p* > 0.05). In addition, the community diversity of CE20 and XE20 was higher than that of LP. In terms of time, the microbial community diversity of CK and LP increased slightly, while CE20 and XE20 decreased slightly. At the same time, the microbial community uniformity (Simpson) was also different for different treatments. Under LP treatment, the distribution of straw silage microorganisms was the most uneven, and the role of dominant microorganisms was the most prominent. However, the distribution of microorganisms in straw raw materials was consistent, and the role of dominant microorganisms was not reflected. Among them, with the increase in silage days, the difference in microbial evenness gradually decreased, and the microbial coverage remained unchanged at 99%.

**Figure 2:**
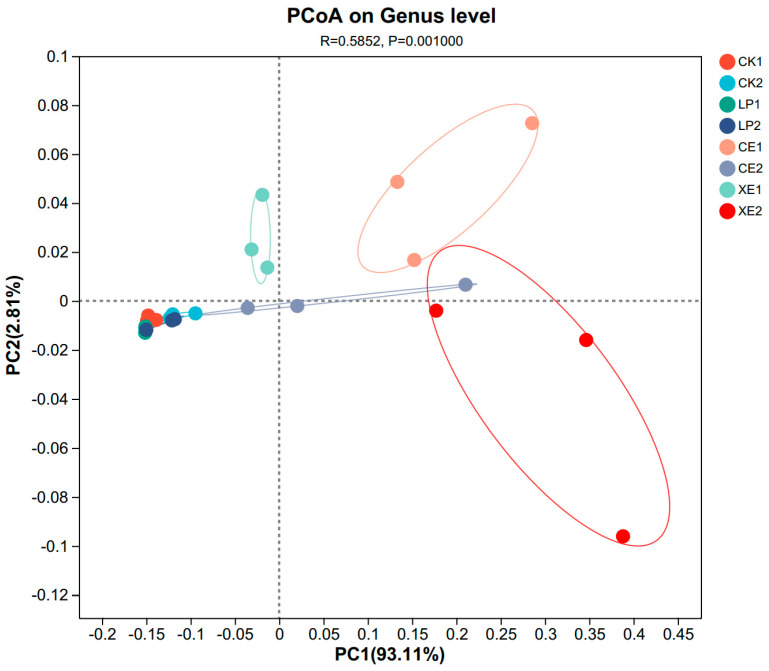
PCoA analysis of different additives and ensiling days broom sorghum straw silage.

The PCoA analysis of broom sorghum stems at the genus level is shown in Figure 2. After 30 days and 60 days of ensiling, the microbial flora of CK and LP did not change significantly, indicating that the microbial community structure of the two treatments did not change significantly during the whole fermentation process. However, the microflora of CE20 and XE20 changed significantly, the use of the two enzyme preparations could not keep the flora structure constant, and XE20 was the most obvious. Among them, the XE20 treatment had the most obvious difference in the flora between 30 d and 60 d of fermentation, and the three repeated flora data after 60 d of fermentation had a large degree of dispersion, indicating that there were different trends in the flora between different replicates.

### 3.5. Microbial Community Phylum and Genus of Broom Sorghum Straw Silage

**Figure 3:**
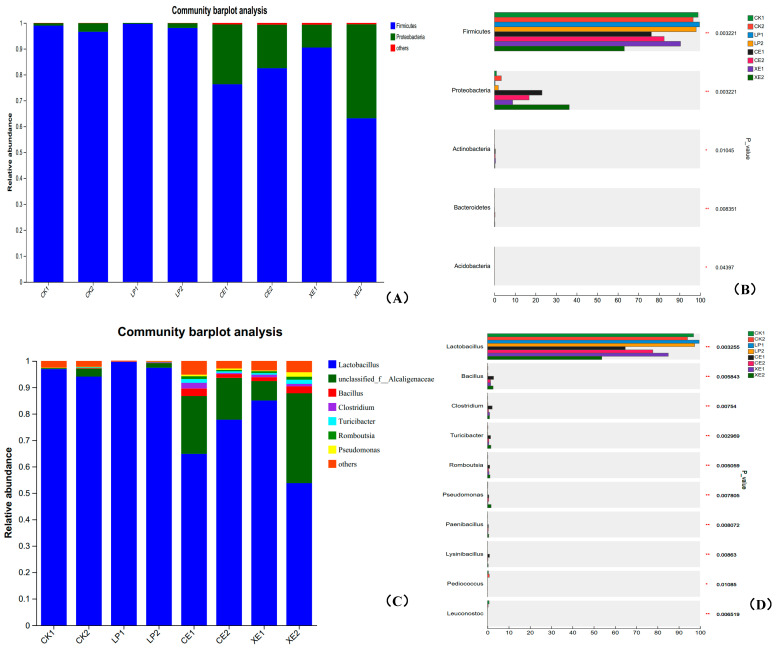
Effects of different treatments on microbial community structure of broom sorghum straw silage at the phylum level. Note: (**A**): microbial species composition at phylum level; (**B**): Differences in microbial species composition between different treatments at the phylum level; (**C**): microbial species composition at genus level; (**D**): Differences in microbial species composition between different treatments at the genus level. 0.01 < *p* ≤ 0.05 marked as *, 0.001 < *p* ≤ 0.01 marked as **.

The effect of microbial community distribution of broom sorghum straw silage is shown in Figure 3. At the phylum level (Figure 3A,B), the abundance of Firmicutes was the highest in the microbial colonies of broom sorghum straw at different fermentation stages, which was the dominant bacterial group, followed by Proteobacteria, and the abundance of other bacteria was low. After 60 days of silage, except for CE treatment, the abundance of Firmicutes decreased and the abundance of Proteobacteria increased in other treatment groups. The order of variation from large to small was LP > CK > CE > XE, while the abundance of other bacteria was not obvious in the fermentation process. With the extension of silage time, the abundance of Firmicutes in CK, LP and XE decreased, and the abundance of Proteobacteria increased.

From the subordinate level of microbial community structure (Figure 3C,D), after 60 days of silage fermentation, the abundance of Lactobacillus was the highest in different treatments of broom sorghum straw, and the abundance of unclassified _ f _ Alcaligenaceae was second only to Lactobacillus. From the perspective of microbial community structure, silage fermentation can change the microbial community, the degree of change is different, and the fermentation effect is also different. CE and XE had a higher abundance of microbial communities than LP and CK, creating an environment more conducive to the growth of unclassified _ f _ Alcaligenaceae and other microorganisms. Compared with silage 30 d, the abundance of Lactobacillus in CE increased significantly, and XE treatment decreased significantly.

### 3.6. The Relationship between Microbial Community and Fermentation Parameters

**Figure 4:**
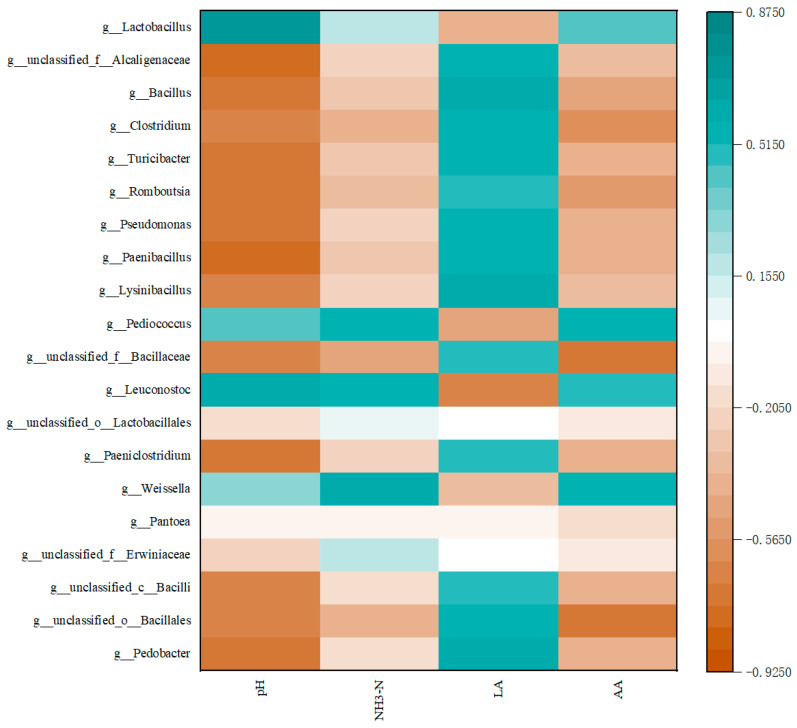
Correlation analysis between microorganisms and fermentation indexes of broom sorghum straw silage.

The correlation between sorghum the straw microbial community (the top 20 dominant species) and fermentation parameters was analyzed by a correlation heat map (Figure 4). The results showed that *Lactobacillus* was positively correlated with the fermentation parameters of sorghum straw (*p* < 0.05), and *Lactobacillus* was significantly positively correlated with pH and LA, indicating that lactic acid bacteria played a beneficial role in the decrease in silage fermentation, pH, and LA production, which could improve the fermentation quality of straw and encourage beneficial microorganisms. *Unclassified _ f _ _ Alcaligenes*, *Bacillus*, *Clostridium*, *Turici-bacter*, *Romboutsia*, *Pseudomonas*, *Paenibacillus* and *Bacillus sphaericus* were positively correlated with straw LA and negatively correlated with other fermentation parameters, indicating that these microorganisms can reduce the loss of straw nutrients by other adverse microorganisms, such as protein, while improving the fermentation quality of straw. The three genera of *Micrococcus*, *Citrobacter* and *Weissella* are positively correlated with NH_3_-N and PA. Although these three microorganisms can promote the formation of acetic acid, they will consume the protein of forage grass and reduce the nutritional quality of the straw.

### 3.7. Metabolic Pathway Prediction of Broom Sorghum Straw Silage at Three Levels

**Figure 5:**
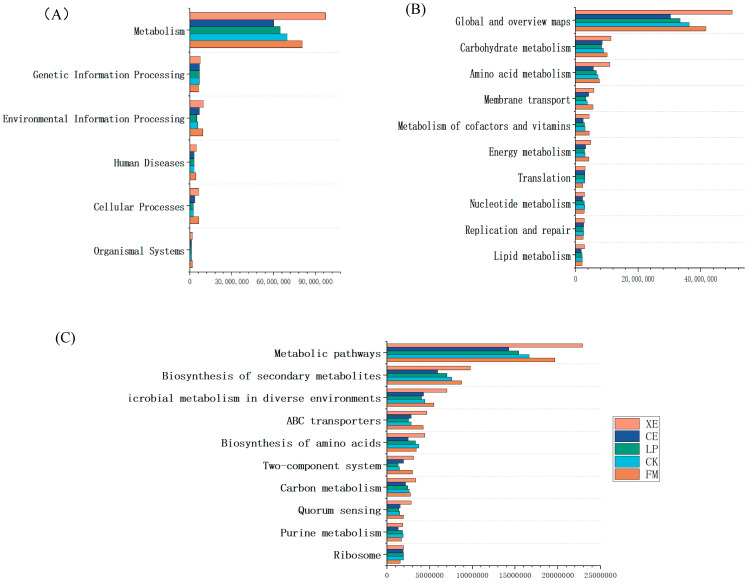
Prediction of 16S rRNA gene function of broom sorghum straw raw materials and silage samples after 60 days of fermentation. Note: (**A**): primary pathway level, (**B**): secondary pathway level, (**C**): tertiary pathway level; FM: straw raw materials; CK: control group; LP: *Lactobacillus plantarum* treatment; CE: cellulase treatment; XE: xylanase treatment.

The 16S rRNA gene function prediction analysis of fresh straw samples and silage samples fermented for 60 days is shown in Figure 5. At the level of primary metabolic pathways, fresh straw samples were consistent with the overall trend of each treatment, mainly enriched in metabolism (Metabolism). There were differences in metabolism (Metabolism) between different treatments. XE treatment could promote this metabolism, while CK, LP and CE treatments inhibited this metabolism. Global and overview, carbohydrate metabolism, and amino acid metabolism are the three major secondary pathway levels of metabolism. The performance of different treatments on the three main secondary metabolic pathways was consistent with the primary metabolic pathway. At the tertiary pathway level, the top 10 metabolic pathways are shown in Figure 5C, and the relative abundance of metabolic pathways is significantly better than other metabolic pathways. After different treatments, the metabolic pathways changed. Among them, XE treatment could promote most metabolic pathways, while the other three treatments inhibited most of the metabolism. After different treatments, the metabolic pathways changed. Among them, XE treatment could promote most of the metabolic pathways, while the other three treatments inhibited most of the metabolism, which should be the key factor causing differences between different treatments.

## 4. Discussion

### 4.1. Nutritional Quality of Broom Sorghum Straw Silage

The stem of broom sorghum is fresh and juicy, palatable, rich in protein, fat and other nutrients before the milky stage, and is easily digested and absorbed by animals [27]. The nutrient content of dry straw is low, and livestock do not like to eat it or even avoid eating it. This is because the harvest time is too late and the storage method is improper, resulting in a serious loss of nutrients [28]. Li et al. [29] found that after silage, the texture of rape straw was soft, the palatability was improved, the nutrient content was increased, and the feeding of livestock was increased. In this study, it was also found that the texture of broom sorghum straw was soft, sour and juicy, and the nutritional value was increased after silage, which was consistent with the results of the former study. This is because in the process of straw silage, under the action of microorganisms and enzymes, the cellulose components in the straw are destroyed; thus, the texture becomes soft, and the carbohydrates produced are used by lactic acid bacteria to produce lactic acid, thus having an acidic odor [30]. Zhao et al. [31] found that the nutritional value of silage corn straw increased by adding fungal preparations to corn straw silage. In this experiment, exogenous additives such as *Lactobacillus plantarum* also increased the nutritional value of broom sorghum straw, indicating that in the straw silage process, the proper use of microbial enzyme additives can promote the fermentation of straw, improve the nutritional value of straw and provide more nutrients for livestock. The life activities of microorganisms are inseparable from nutrients. During the silage process of broom sorghum straw, the life activities of microorganisms reduce the dry matter of straw. With the use of cellulase and xylanase, more fiber components in broom sorghum straw were degraded, and the content of WSC increased, which was consistent with the results of Yujie Zhai et al. [32]. In conclusion, the use of the silage process and additives can improve the nutritional quality and palatability of straw and increase the feeding interest of livestock.

### 4.2. Fermentation Quality of Broom Sorghum Straw Silage

The decrease in pH value is considered to be an important indicator reflecting the fermentation process of silage, and the accumulation of NH_3_-N during silage is generally considered to be an indicator of protein degradation [33]. The dynamic changes in pH and NH_3_-N in broom sorghum straw silage indicated that different additive treatments may affect the fermentation process. The results of this experiment showed that the fermentation of broom sorghum straw treated with different additives could effectively reduce the pH of silage, and the treatment effect of cellulase and xylanase was better than that of lactic acid bacteria. Li et al. [30] found that the addition of lactic acid bacteria and cellulase during the mixed silage of amaranth and rice straw could significantly reduce the pH value and NH_3_-N content and increase the LA content. In this study, it was also found that the LA content of silage added with cellulose was significantly higher than that of other treatments. In the study of additives for woody plant silage, Du et al. pointed out that the use of cellulase can destroy the connection bond between the cell wall and the fiber material in the straw, resulting in more nutrients being exposed and increasing the fermentation of lactic acid bacteria. The substrate produces more LA and reduces the effect of other microorganisms on silage quality in the early stage of fermentation [34]. With the increase in silage days, there was no significant difference in pH and NH_3_-N between the 30 d sample and the 60 d sample, which indicated that the silage of broom sorghum straw entered a stable state after 30 days of fermentation. In xylanase treatment, the increase in LA content may be related to the higher WSC content in the early stage, and the microorganisms could not be fully utilized in the early stage of fermentation. The use of additives is helpful for the degradation of straw, promotes the growth of lactic acid bacteria, rapidly reduces the pH, reduces the threat of other microorganisms, and ensures the normal fermentation process.

### 4.3. Microbial Community of Broom Sorghum Straw Silage

The results of high-throughput sequencing technology showed that the microbial coverage of broom sorghum straw samples was above 0.99 in different treatments, indicating that most bacteria in broom sorghum straw were detected by high-throughput sequencing technology. The study found that compared with FM, the shannon index of each treatment decreased, which may be because most of the microorganisms in the fermentation process could not tolerate the closed and anaerobic acidic environment, and were replaced by lactic acid bacteria, so the observed species decreased [35]. At the same time, between different treatments, the diversity of microbial communities under CE and XE treatments was also significantly increased compared with CK and LP treatments. This is because the use of the two enzymes releases more nutrients from sorghum straw, which promotes the growth of other microorganisms [34,36,37]. After ensiling fermentation, the bacterial community mainly evolved into Firmicutes, and the anoxic and acidic environment was beneficial to the growth of Firmicutes and inhibited the growth of other bacteria [38]. At the genus level, unclassified _ f _ Alcaligenaceae was found in all treatments. Relevant studies have found that the use of exogenous additives in the anaerobic fermentation system of silage can effectively degrade lignin degradation derivatives such as ferulic acid [39,40]. Therefore, the use of cellulase is better than other additives in the process of broom sorghum straw silage.

### 4.4. Silage Microbial Gene Function Prediction

PICRUSt2 can be used to predict the function of the KEGG database based on 16 S sequencing data, including the metabolism and genetic information of bacteria [41]. This study shows that at the level of the first pathway, metabolism is the main metabolic pathway, indicating that the silage fermentation process is essentially caused by microbial metabolism. Microorganisms use fermentation substrates to meet their own growth and reproduction and also convert some organic matter into new metabolites [17]. At the second level, this study analyzed global and overview maps and found that there were differences between different treatments. These differences are in carbohydrate metabolism, amino acid metabolism, nucleotide metabolism, energy metabolism, cofactors and vitamin metabolism. Related studies have pointed out that these metabolic pathways play an important role in the formation of silage quality [42]. In order to further understand the related metabolism, we carried out a study at the third level. Silage is the use of lactic acid bacteria to ferment water-soluble carbohydrates to produce lactic acid, thereby preserving and improving the nutritional quality and palatability of forage. With the fermentation process of lactic acid bacteria, the synthesis of many secondary metabolites is also carried out. This study also found that there were differences in metabolic pathways among different treatments, which were mainly manifested in the biosynthesis of secondary metabolites, microbial metabolism in diverse environments, and so on. This may be the decisive factor for the difference in nutritional quality of broom sorghum straw under no treatment. Bao et al. [43] found that the biosynthesis of plant secondary metabolites affected the terpenoids and yellow color in silage. The volatile compounds produced by microbial metabolism are an important factor in changing the flavor of raw materials. In the fermentation process of rapeseed meal, the content of pyrazine compounds is greatly increased during the fermentation process, followed by volatile substances such as butyric acid and phenylpropionitrile [44].

## 5. Conclusions

This study evaluated the effects of Lactobacillus plantarum, cellulase and xylanase additives and fermentation time on fermentation parameters, chemical composition and bacterial community of sorghum straw silage. The results showed that the quality of straw silage increased with the prolongation of silage time. At the same time, the use of Lactobacillus plantarum, cellulase and xylanase improved the silage characteristics of sorghum straw by regulating the bacterial community. Compared with straw raw materials, the CP content in silage straw increased significantly, with LP treatment having the best effect and XE treatment having the worst effect. In addition, compared with the direct fermentation of straw, the use of three additives can increase the abundance of Lactobacillus plantarum, reduce the pH value, and quickly inhibit bad microorganisms such as Escherichia coli. Compared with Lactobacillus plantarum, cellulase can increase the content of WSC in silage and increase the concentration of LA and AA. Therefore, cellulase is more suitable for the silage of sorghum straw. It is recommended to add cellulase to the silage of sorghum straw with a standard of 20U/g FM and open the package after 60 days of sealed fermentation. In the future, research on the composition of fiber components in broom sorghum and the degradation law of fiber components in the micro-storage process can be strengthened to further improve the utilization efficiency of broom sorghum straw.

## Figures and Tables

**Table 1:** Nutrient composition and microbial count of broom sorghum straw raw materials.

Item	Specimen
Dry Matter DM (g/kg FM)	926 ± 11.27
Crude Protein CP (g/kg DM)	35.63 ± 1.67
Neutral Detergent Fiber NDF (g/kg DM)	853.22 ± 4.96
Acid Detergent Fiber ADF (g/kg DM)	613.97 ± 15.26
Water Soluble Carbohydrates WSC (g/kg DM)	31.60 ± 3.74
Lactic acid bacteria (Log_10_ cfu/g FM)	6.53 ± 1.08
Saccharomyces (Log_10_ cfu/g FM)	7.12 ± 0.67
Colibacillus (Log_10_ cfu/g FM)	6.32 ± 1.34
Aerobe (Log_10_ cfu/g FM)	8.57 ± 0.32
Fungus (Log_10_ cfu/g FM)	2.34 ± 0.25

FM, fresh matter; DM, dry matter.

**Table 2:** Effects of different additives and ensiling days on nutritional quality of broom sorghum straw silage.

	Time	CK	LP	CE10	CE20	XE10	XE20	SEM	T	D	T × D
DM (g/kg FM)	30	423.27 ^Ab^	416.74 ^Abc^	419.00 ^Abc^	416.08 ^Ac^	437.97 ^Aa^	418.46 ^Abc^	0.00	<0.0001	<0.0001	0.3282
	60	419.22 ^Ab^	408.08 ^Bc^	409.30 ^Ac^	406.93 ^Bc^	436.78 ^Aa^	411.88 ^Abc^				
CP (g/kg DM)	30	19.72 ^Bab^	20.86 ^Ba^	17.23 ^Bbc^	22.35 ^Ba^	16.73 ^Bc^	20.69 ^Ba^	0.10	<0.0001	<0.0001	0.0022
	60	32.29 ^Aa^	33.95 ^Aa^	30.79 ^Aa^	31.08 ^Aa^	23.81 ^Ab^	26.01 ^Ab^				
WSC (g/kg DM)	30	11.85 ^Ac^	17.16.5 ^Ac^	31.06.9 ^Ab^	31.35 ^Ab^	37.48 ^Aab^	43.02 ^Aa^	0.00	<0.0001	<0.0001	0.0006
	60	12.14^Ab^	12.53^Ab^	16.94^Ab^	31.08^Aa^	14.17^Bb^	33.43^Aa^				
ADF (g/kg DM)	30	601.62 ^Aa^	580.83 ^Ab^	541.01 ^Ac^	543.28 ^Ac^	560.76 ^Abc^	560.72 ^Abc^	0.00	<0.0001	0.1723	0.5128
	60	567.03 ^Ba^	571.05 ^Aa^	520.65 ^Ac^	530.6 ^Abc^	541.98 ^Ab^	530.51 ^Bbc^				
NDF (g/kg DM)	30	846.85 ^Aa^	827.56 ^Aa^	798.63 ^Ab^	780.15 ^Ab^	787.25 ^Ab^	798.63 ^Ab^	0.01	<0.0001	<0.0001	0.5890
	60	790.52 ^Bab^	795.94 ^Aa^	758.55 ^Ab^	755.67 ^Bb^	757.98 ^Ab^	763.32 ^Bab^				

Note: (1) DM: dry matter; CP: crude protein; WS: water-soluble carbohydrates; ADF: acid detergent fiber; NDF: neutral detergent fiber. (2) Different lowercase letters of peer data indicated that the difference between different treatments of the same silage days was significant (*p* < 0.05). (3) Different uppercase letters of the same column data represent significant differences between different silage days under the same treatment (*p* < 0.05), and the same uppercase letters represent no significant difference between different treatments. (4) T: different additive treatment; D: different days of silage; T × D: interaction between additive treatment and silage days; SEM, standard error.

**Table 3:** Effects of different additives and ensiling days on fermentation characteristics of broom sorghum straw silage.

	Time	CK	LP	CE10	CE20	XE10	XE20	SEM	T	D	T × D
pH	30	4.24 ^Aa^	4.16 ^Aa^	3.85 ^Ab^	3.85 ^Ab^	3.87 ^Ab^	3.85 ^Ab^	0.04	<0.0001	0.9877	0.9811
	60	4.38 ^Aa^	4.07 ^Ab^	3.86 ^Ab^	3.87 ^Ab^	3.77 ^Ab^	3.78 ^Ab^				
NH_3_-N (g/kg FM)	30	0.13 ^Ba^	0.05 ^Ab^	0.02 ^Bb^	0.04 ^Ab^	0.03 ^Ab^	0.02 ^Ab^	0.15	<0.0001	0.0009	0.0010
	60	0.39 ^Aa^	0.06 ^Ab^	0.05 ^Ab^	0.06 ^Ab^	0.07 ^Ab^	0.05 ^Ab^				
LA (g/kg FM)	30	10.71 ^Ac^	14.04 ^Abc^	18.79 ^Ba^	19.28 ^Aa^	16.98 ^Bab^	15.38 ^Bab^	0.71	<0.0001	0.0006	0.7536
	60	11.53 ^Ab^	19.91 ^Aa^	23.36 ^Aa^	23.79 ^Aa^	20.55 ^Aa^	19.02 ^Aa^				
AA (g/kg FM)	30	3.38 ^Aa^	1.16 ^Ab^	0.20 ^Bb^	0.28 ^Ab^	0.36 ^Ab^	0.03 ^Bb^	0.27	<0.0001	0.0338	0.7281
	60	3.99 ^Aa^	3.03 ^Aab^	0.78 ^Abc^	0.64 ^Abc^	0.50 ^Ac^	0.98 ^Abc^				
PA (g/kg FM)	30	12.01 ^Ba^	7.74 ^Bbc^	8.60 ^Abc^	6.91 ^Bbc^	10.53 ^Aab^	6.29 ^Bc^	0.59	<0.0001	<0.0001	0.0002
	60	18.06 ^Aa^	15.04 ^Ab^	11.28 ^Ac^	9.64 ^Acd^	11.35 ^Ac^	8.19 ^Ad^				

Note: (1) NH_3_-N: ammonia nitrogen; LA: lactic acid; AA: acetic acid; PA: propionic acid. (2) Different lowercase letters of peer data indicated that the difference between different treatments of the same silage days was significant (*p* < 0.05). (3) Different uppercase letters of the same column data represent significant differences between different silage days under the same treatment (*p* < 0.05), and the same uppercase letters represent no significant difference between different treatments. (4) T: different additive treatment; D: different days of silage; T*D: interaction between additive treatment and silage days; SEM, standard error.

## Data Availability

The raw sequence data were uploaded to the NCBI archive of sequence reads under study record number PRJNA1141159.
